# The County Council as a Health Authority

**Published:** 1907-03-23

**Authors:** 


### THE COUNTY COUNCIL AS A HEALTH AUTHORITY.

### OFFENSIVE TRADES.

It is very little known how much the amenity of our lives depends on the control exercised by the County Council over offensive businesses. If there were no control exercised, no sanction needed before setting up a slaughter-house or a soap-boiler's factory, a whole neighbourhood might be rendered uninhabitable through the selfishness of one man. Happily no man is thus allowed to annoy his neighbours, and certain trades, classed as offensive, are licensed annually to be carried on only in localities where the nuisance they create is not too great. The slaughter-houses far outnumber the other businesses, but with the changing conditions of our meat supply they grow fewer in number every year. In 1905 the number of premises licensed for this purpose was 318, as against 333 in the previous year, 346 in 1903, and so on, in a steadily ascending scale. In 1892 licenses were granted for 537 premises, which shows how great the decrease has been. The premises are inspected frequently, and most of them seem to come out of the ordeal well. In many districts there was no need to take any action, and in many only a single notice was served. Hammersmith, however, enjoys a bad pre-eminence. It has twelve slaughter-houses, and it was found necessary to serve twenty-nine notices, though none of the slaughter-houses was taken off the register. Probably the whole twelve premises are not in fault, and it might be a good thing, both for those who keep the law and for the public, if those who ignore the sanitary regulations were removed from the register.

The other trades which are designated "offensive" include fat-melters, tripe-boilers, glue and size manufacturers, knackers, fellmongers, bone-boilers, manure manufacturers, soap-boilers, tallow-melters, gut manufacturers, gut-scrapers, and animal charcoal manufacturers. It can hardly be questioned that they fully deserve the cognomen. It is more open to question whether some other businesses should not be included in the same category. There is, for example, the fried fish trade. In one case a fried fish seller was summoned because the smell from his premises rose to the upper floors of the building, where a tailor's workshops were situated, and impregnated some uniforms that were being made there. In this case a judge's order was made requiring that the nuisance should be abated, and the business was discontinued. As fried fish shops are to be found in many of the poorer streets of London, and have a far-reaching and disagreeable smell, it is likely enough that this was not the only case in which the odour caused a nuisance, although it was the only one complained of. If this business were set down among offensive trades, it would be within the power of the County Council, even when no complaint of a nuisance was made by neighbours, to make regulations not only as to the places where the business could be carried on, but also as to the frying furnaces and the methods of getting rid of offensive vapours. As a matter of fact, many furnaces are defective in these respects.

Although the odours from the frying fish are often disagreeable, they cannot be regarded as dangerous to health. There is far more risk to public health from the quality of the fish sold, and the difficulty of disposing promptly and satisfactorily of the offal and refuse. It is possible that stricter regulations as to the size of fish that may be sold for human consumption would be helpful in this connection. In the summer, from April to July, a large number of small and immature plaice are caught and brought to market, and, being cheap, they are largely bought by the fried fish merchants, whose customers are practically all of the poorer class. While large fish are gutted as soon as they are caught, in order to preserve them, these small fish are sent to market as they are caught, because of the time and trouble involved in cleansing them, and thus when they reach the retailer they are often more or less decomposed. If there were some law about the size of fish that might be sold, there would be a restriction on the using of these small and often unwholesome fish. "Chip potatoes" are a frequent concomitant of fried fish, and it seems that these potatoes are often fried without previous peeling, and also that diseased potatoes are not as carefully excluded as might be. If the trade of fish frying were put under regulation, better control would probably be exercised in respect of the materials used, which would make for public health, and it would be practicable to insist on certain makes of furnace, with a hood and ventilation, which would get rid of the offensive vapours caused by the frying, and thus make for public comfort. Where the fish are cleansed on the premises it would be necessary also to see that the arrangements were such as to secure adequate washing of the fish before frying, and a satisfactory method of getting rid of the offal.

Another business, and one which is comparatively little known, which might be regarded as offensive is that of a fish-curer. The bulk of this trade is done in Scotland and in the North of England, but there are about 550 curing places within the limits of London. The majority of these are small places, with barely three smoke-holes each on an average. The fish—herring or haddock—are cleansed, rubbed with salt, and suspended over slow-burning fires of pine, elm, and oak sawdust for seven or eight hours. In some instances, however, herrings are merely air-dried in shop windows or in sheds or buildings in the rear of shops. The County Council report goes on to state: "It has been said that some street costers cure herrings by air-drying in their living or sleeping rooms, but no single instance of this was actually found; in one instance, however, curing was being carried on in a horse stable at Hackney Wick." This fact will scarcely increase the sale of bloaters if the public gets to know of it. Certainly the record of these curing-places is not pleasant reading. In 10 per cent. of the places the gutting and cleansing is done in open back-yards, often defectively paved, and in 60 per cent. in temporary wooden shelters or sheds not fitted with impervious surfaces, and in some cases totally unsuitable for the conduct of the business. In three cases basements, in sixteen cases stables or places in aerial communication with stables were used; in twenty-eight cases a water-closet was inside or was in aerial communication with the curing-place; in three cases fowls or other animals were kept; and at one wholesale place no fewer than five cats were kept on the premises. Where the walls were unprovided with impervious smooth surfaces they were generally caked and splashed with decomposing fish-scales and filth. The gutting-benches were generally constructed of wood, absorbing filthy liquids, and so giving rise to nuisance. Facts like these make one wonder that this business has not long since been scheduled as an offensive trade, and make one desire to see the strong arm of the County Council extended still further than it is.

---

The proprietors of Oxo announce a new coupon scheme. In exchange for Oxo coupons the firm supplies a portrait enlargement from the customer's own photograph. These productions are beautifully finished examples of art photography, and measure 23 by 19 inches. Full particulars may be obtained on application to Oxo Portrait Department, 4 Lloyd's Avenue, London, E.C.

